# Supplementary material for: Diet-induced steatohepatitis does not cause heart failure with preserved ejection fraction in male middle-aged C57BL/6N mice
Source: PLoS One. 2025 Dec 29;20(12):e0339642. doi: 10.1371/journal.pone.0339642 (PMC12747382; doi:10.1371/journal.pone.0339642)
Supplement: S1 Table — (PDF) [file pone.0339642.s001.pdf]

S1 Table 1. Primers used in this study

Species	Gene	Forward	Reverse
Mouse	*ColA1*	CCCAGCCGCAAAGAGTCTAC	GGGTTTCCACGTCTCACCAT
Mouse	*Icam1*	CAGTCCGCTGTGCTTTGAGA	AGGGTGAGGTCCTTGCCTAC
Mouse	*Il1b*	TGTGCAAGTGTCTGAAGCAGCTA	TCAAAGGTTTGGAAGCAGCCCT
Mouse	*Ccl2*	GTTGGCTCAGCCAGATGCA	AGCCTACTCATTGGGATCATCTTG
Mouse	*Nppa*	CACAGATCTGATGGATTTCAAGA	CCTCATCTTCTACCGGCATC
Mouse	*Nppb*	GTCAGTCGCTTGGGCTGT	CAGAGCTGGGGAAAGAAGAG
Mouse	*Myh6*	CTCTGGATTGGTCTCCCAGC	GTCATTCTGTCACTCAAACTCTGG
Mouse	*Myh7*	GAGGAGAGGGCGGACATT	ACTCTTCATTCAGGCCCTTG
Mouse	*Gapdh*	CAGGTTGTCTCCTGCGACTT	GGCCTCTCTTGCTCAGTGTC
