# Supplementary figures and images for: Diet-induced steatohepatitis does not cause heart failure with preserved ejection fraction in male middle-aged C57BL/6N mice
Source: PLoS One. 2025 Dec 29;20(12):e0339642. doi: 10.1371/journal.pone.0339642 (PMC12747382; doi:10.1371/journal.pone.0339642)

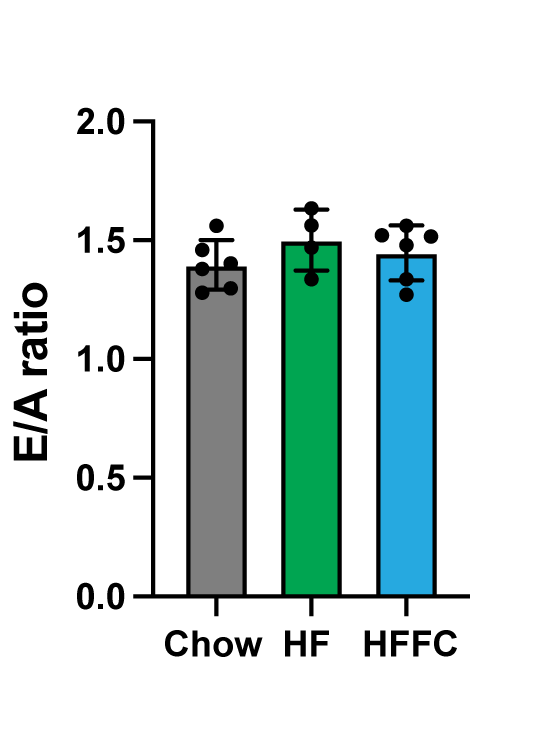

Supplement: S1 Fig — Early to late diastolic transmitral flow velocity ratio (E/A). N = 6. (TIF) [file pone.0339642.s002.tif]

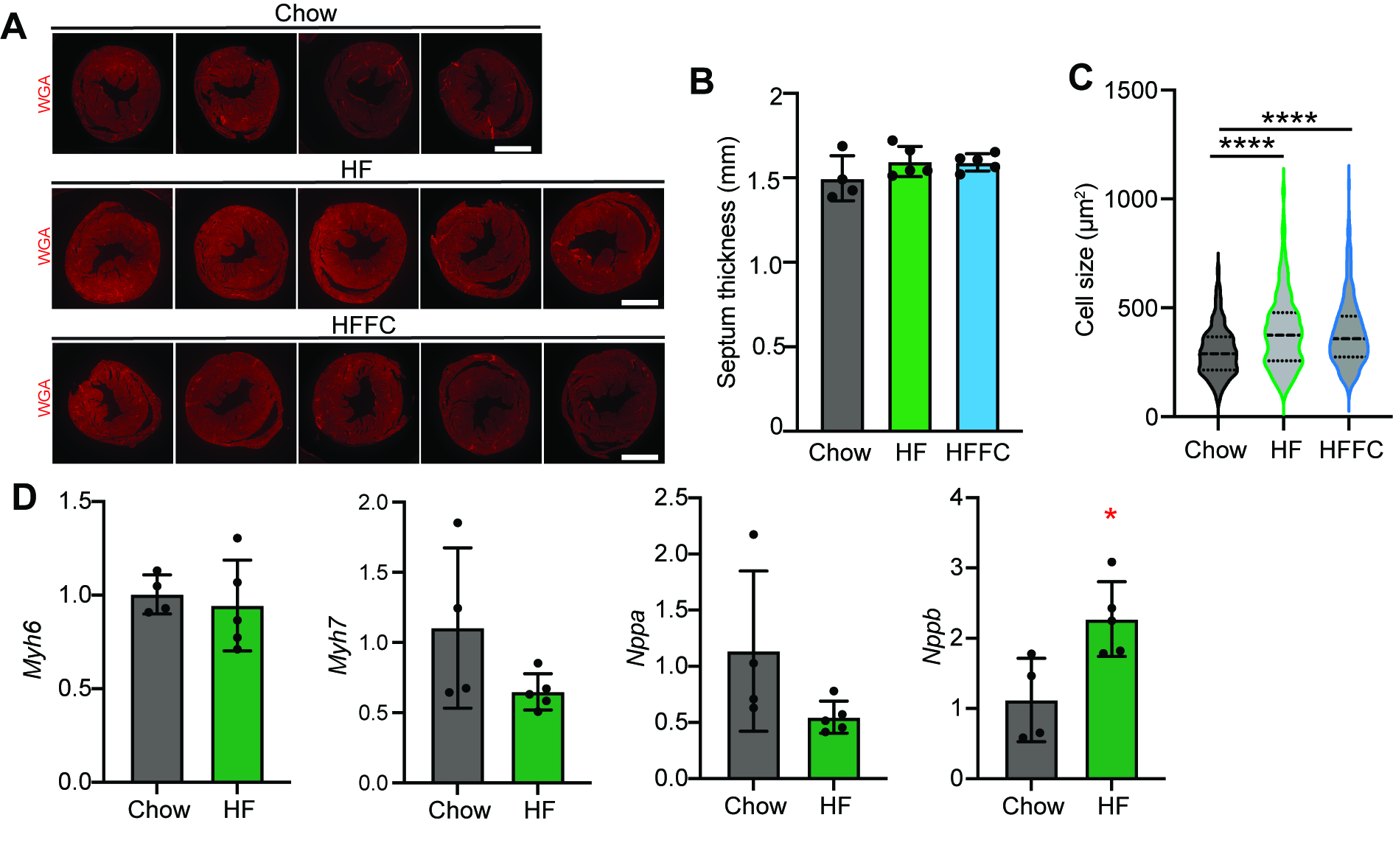

Supplement: S2 Fig — A. WGA-stained cardiac cross sections. Scale, 2 mm. B. Cardiac septum thickness measurement. C. Papillary muscle cross-sectional area measurement. Chow diet group, 200 cells from 4 hearts were measured. For each group of the high-fat diet (HF) and HFFC diet-treated mice, 250 cells from 5 hearts were measured. Kruskal-Wallis test, ****, p < 0.0001. D. Quantitative PCR analysis of heart gene expression. Student t-test, *, p < 0.05. N = 5. (TIF) [file pone.0339642.s003.tif]

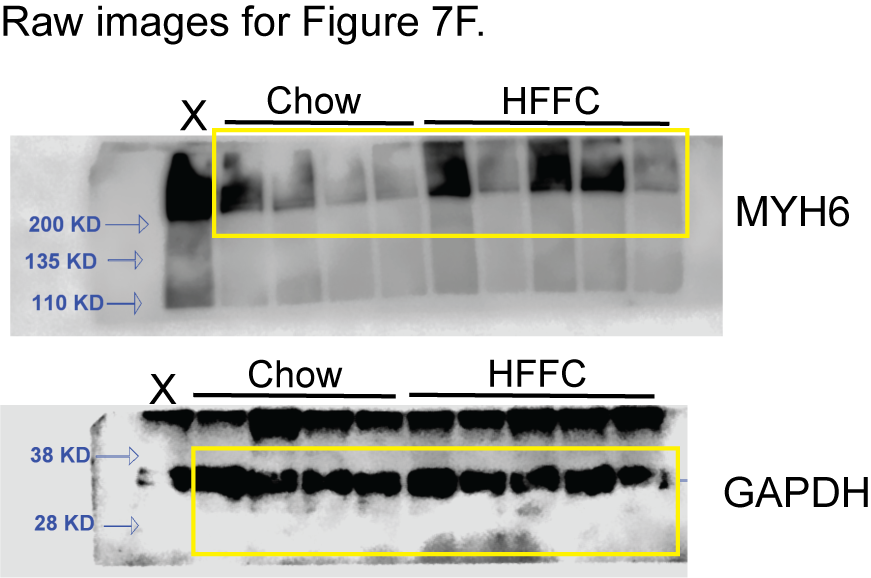

Supplement: S1 Raw Images — (TIF) [file pone.0339642.s004.tif]
